# Toward an operational definition of Artificial Intelligence for health care informatics: a Delphi survey

**DOI:** 10.1093/haschl/qxaf243

**Published:** 2025-12-23

**Authors:** Carolyn Sun, Shakib Hossain, Shannon L Harris

**Affiliations:** Hunter-Bellevue School of Nursing, Hunter College, New York, NY 10010, United States; NewYork-Presbyterian Columbia Irving Medical Center, New York, NY 10032, United States; Virginia Commonwealth University, Richmond, VA 23284, United States

**Keywords:** Artificial Intelligence, consensus, definition, Delphi survey

## Abstract

The proliferation of Artificial Intelligence (AI) technologies, fueled by advancements in computational power and generative models, is rapidly reshaping healthcare delivery and research. However, the absence of a standardized definition of AI impedes regulatory development, confounds public discourse, and hinders clinical adoption. This study provides clarity for AI developers and users in terminology surrounding the topic, which will ultimately assist in mitigating risks to patients and the public. Utilizing a multiphase Delphi method involving international informatics experts, we synthesized existing definitions and facilitated consensus on an operational definition of AI tailored to healthcare contexts. Our findings aim to establish a foundational framework to guide ethical governance, promote funding alignment, and optimize AI integration in clinical settings.

## Teaser

Artificial Intelligence (AI) is transforming health care—from diagnostic imaging and predictive analytics to virtual assistants and clinical decision support. Yet a fundamental problem remains: What exactly counts as “AI”? Despite the surge of AI-enabled tools, the lack of a shared definition in healthcare informatics continues to impede policy coherence, ethical oversight, and clinical adoption. In this International Delphi Consensus Study, researchers convened informatics experts from medicine, nursing, and policy to agree on an operational definition of AI specifically for health care informatics. Participants emphasized clarity over complexity—favoring a concise description that removes anthropomorphic language such as “reasoning” or “deciding,” and highlights AI as a human-designed, data-driven system. The resulting consensus definition—endorsed by >80% of respondents—provides a common foundation for regulators, clinicians, and developers to identify what qualifies as AI in healthcare informatics. It marks a critical step toward consistent governance and public understanding, helping distinguish between algorithmic decision-support tools and truly autonomous systems. As health systems increasingly rely on AI to guide care, words matter. A clear, expert-endorsed definition is more than semantics—it's a prerequisite for transparency, accountability, and trust in the digital future of health care.

## Introduction

The rapid growth of Artificial Intelligence (AI) in health care informatics—the interdisciplinary field that uses information technology and data science to manage information from healthcare (eg, electronic health record data) to make improvements—has outpaced regulatory, ethical, and research frameworks. Despite the proliferation of AI tools in clinical settings, the absence of a shared operational definition continues to impede governance, policy alignment, and clinical adoption.^1^ Ambiguity around what constitutes AI complicates oversight, contributes to inconsistent reporting, and weakens public trust. To address this foundational gap, we conducted an International Delphi Consensus Study to identify a concise, expert-driven definition of AI tailored for health care informatics.

## Methods

The Delphi process was introduced at the 2024 American Medical Informatics Association (AMIA) Annual Symposium and was conducted via iterative online surveys. Attendees of the conference were recruited via announcements at topically related presentations, flyers, and personal invitations from the researchers. Participants represented a broad range of expertise including informatics, medicine, nursing, data science, ethics, and policy (Appendix Table S1). The first round gathered open-ended feedback on the European Commission's 2019 “AI Watch” definition.^2^ Responses were qualitatively analyzed to identify five major themes (Table 1). The second round recruited participants from the first round and was also posted on the internal social media board for AMIA members. This round quantified agreement with proposed revisions using a five-point Likert scale. Because Delphi consensus thresholds vary widely (often 50%-75%), we used ≥50% to identify areas of shared agreement across a heterogeneous informatics panel.^3^ This study was approved by the authors' institutional review board. The full methods are presented in the Appendix.

**Table 1.** Delphi results.

Major themes			
Major category	Round 1 (Agree) *N* = 34	Round 1 % of suggestions*	Round 2 (Agree) *N* = 63
Make the definition more concise	34	55.7%	51
Expand on definition	9	14.8%	16
Leave as is	6	9.8%	19
Change definition completely	9	14.8%	22
Do not try to define AI	3	4.9%	6

Round 2 Delphi Consensus Results (top items), *n* = 63			
Theme or proposed revision	Participants *n* (agree + somewhat agree)	% (Agree + somewhat agree)	Consensus outcome
**Overarching theme**			
Make the definition more concise	1	81.0%	Consensus achieved
**Subthemes**			
Remove “given a complex goal”	42	66.7%	Consensus achieved
Remove the term “and/or hardware”	37	58.7%	Consensus achieved
Remove “perceiving their environment”	36	57.1%	Consensus achieved
Simplify the number of conditions	35	55.6%	Consensus achieved
Delete anthropomorphic terms	32	50.1%	Consensus achieved

*This is the proportion of all suggestions made within this category (these are the broad themes), not the number of participants. Participants could contribute multiple suggestions, and each could fall into more than one category. Therefore, categories are not mutually exclusive, and the percentages reflect the proportion of total coded suggestions rather than the proportion of respondents (*n* = 34, 63, Rounds 1 and 2, respectively). As a result, percentages may sum to >100%.

## Results

In Round 1 (*N* = 34), participants emphasized the need for conciseness and clarity, and in more specific suggestions under the major themes, recommended the removal of anthropomorphic language such as “perceiving,” “deciding,” or “reasoning.” They also advised eliminating unnecessary references to hardware and simplifying the description of goals and functions.^4^ In Round 2 (*N* = 63), 81.0% of participants agreed the definition should be made more concise, indicating strong consensus. Within this overarching category, the subcategories with strongest consensus items included removing the phrases “given a complex goal” (66.7%), “perceiving their environment” (57.1%), and anthropomorphic terms (50.1%). More than half of respondents disagreed with expanding the definition or abandoning the effort to define AI altogether. Participant characteristics did not vary significantly by round (Appendix Table S1).

Integrating these consensus-driven revisions resulted in the following definition:

**Artificial intelligence (AI) systems are systems designed by humans that act in the physical or digital dimension through the acquisition of structured or unstructured data using complex algorithms or neural networks to construct information, knowledge, and recommendations to achieve the given goal**.

## Discussion

This concise definition eliminates anthropomorphic and overly technical terms while preserving conceptual rigor. It emphasizes AI as human-designed, data-driven, and purpose-oriented—key elements identified as essential for policy and practice alignment within healthcare informatics.^1^ Participants noted that this phrasing avoids framing AI as autonomous or sentient, which is critical for managing both public and clinical expectations.^4^ For example, currently, there are no standard competencies within nursing (the largest sector of the healthcare workforce) regarding AI. To implement such competencies or policies, a fundamental step would be an operational definition of AI for health informatics. The resulting clarity of an operational definition supports consistent communication among developers, regulators, and healthcare professionals.

A standardized definition of AI carries several implications. First, it enables policymakers and regulators to delineate which technologies warrant ethical and safety oversight.^5^ Clear scope is a prerequisite for implementing transparency, accountability, bias, and privacy standards across health systems. Second, clarity benefits researchers and funding bodies by establishing a coherent conceptual basis for identifying and evaluating AI-related work in healthcare informatics. Third, informaticists in health organizations can better assess, adopt, and communicate about AI tools when terminology is consistent and unambiguous.^6^ As prior work notes, inconsistent definitions have constrained evaluation frameworks and slowed AI integration across clinical environments.^1,5^

Because there is so little mandated training in AI and informatics in healthcare, in regard to health informatics, clinicians are largely operating from a lay perspective; definitional precision can improve understanding. AI remains popularly associated with humanoid robots and science fiction narratives, creating misconceptions that fuel distrust.^4^ By focusing on data, algorithms, and human design, the consensus definition differentiates AI from autonomous systems and underscores its bounded functionality. This helps align lay perception with scientific reality, mitigating exaggerated expectations and concerns.^2,4^

The Delphi findings reveal that healthcare informatics experts overwhelmingly prefer definitions that are concise, flexible, and accessible. Such definitions can evolve with technological progress while maintaining core principles that emphasize human agency and data-driven processes.^1,5^

### Limitations

This study was limited to professionals working in healthcare informatics, thereby limiting generalizability to other fields. However, within healthcare informatics, the diversity of respondents—spanning informatics, nursing, medicine, and ethics—enhances confidence in the results for healthcare informaticians. Future research should test how this consensus definition performs in applied contexts, including AI regulation, health policy development, and educational curricula.^6^

## Conclusion

Establishing definitional clarity is a foundational step toward coherent governance of AI in healthcare informatics. It provides a common reference point for evaluating safety, reliability, and ethical implications of emerging technologies.^5^ By advancing an expert consensus definition, this study contributes to global efforts to build trust, transparency, and accountability in AI-enabled health systems.^1,6^

## Supplementary Material

qxaf243_Supplementary_Data
