# Supplementary material for: Toward an operational definition of Artificial Intelligence for health care informatics: a Delphi survey
Source: Health Aff Sch. 2025 Dec 23;4(1):qxaf243. doi: 10.1093/haschl/qxaf243 (PMC12778320; doi:10.1093/haschl/qxaf243)
Supplement: qxaf243_Supplementary_Data [file qxaf243_supplementary_data.zip › appendix_00522R1.docx]

**APPENDIX**

**Methods**

**Survey Distribution and Data Collection.** This study used a Delphi survey (a multi-round, iterative survey) to gain consensus on a definition of AI among a group of healthcare informatics experts. The survey was introduced at the 2024 American Medical Informatics Association (AMIA) Annual Symposium which was held November 9-13, 2024. Each survey round was designed to take approximately 15 minutes to complete, and we expected a minimum of two and a maximum of three rounds. We opened the first survey round at the beginning of the conference (November 9, 2024), allowing immediate participation through a QR code on a recruitment flyer. Additional recruitment was done through social media posts (AMIA Connect, an internal platform for AMIA members, and LinkedIn from the PI’s account). Consent was obtained at the beginning of each survey round via Qualtrics. We aimed to recruit at least 30 participants per round. The first round was closed upon data saturation (no new themes or insights emerging). For subsequent rounds, results were compiled, and the survey distributed to participants who provided an email address in the previous round. Updates were also posted on AMIA Connect and through social media to gain additional participants. Participants received a $15 Amazon or Visa e-gift card for completing each survey round (n = maximum $45 incentive).

**Participant Recruitment.** We strived to recruit participants with diverse backgrounds in AI, policymaking, academia, and industry, emphasizing diversity in expertise and viewpoints by announcing the study at various meetings and workshops at AMIA representing a broad range of those working in healthcare. Adult attendees (age 18 or greater) who work with or in Artificial Intelligence were eligible to participate.

**Survey.** In each round, the first block of the survey obtained the participants' consent and collected basic demographic information (race, ethnicity, gender, area of expertise, years of experience, name, place of employment, and email address).

In the first round of the survey, participants were asked an open-ended question informed by the literature as follows:

*“The purpose of this study is to come to a current consensus on the operational definition of Artificial Intelligence within healthcare. The following definition was proposed by AI Watch (the full report may be read here):*

*"Artificial intelligence (AI) systems are software (and possibly also hardware) systems designed by humans that, given a complex goal, act in the physical or digital dimension by perceiving their environment through data acquisition, interpreting the collected structured or unstructured data, reasoning on the knowledge, or processing the information derived from this data, and deciding the best action(s) to take to achieve the given goal. AI systems can either use symbolic rules or learn a numeric model, and they can also adapt their behavior by analyzing how the environment is affected by their previous actions." (AI HLEG, 2019a)*

*Is this definition adequate for AI within healthcare? If not, what do you suggest should be added or removed? For example, are there aspects specific to healthcare that should be added? Any additional thoughts?*

**Analysis of Survey Data.** In round one, open-ended responses were qualitatively assessed by the research team to gain nuanced insights into participants' viewpoints. Responses from round one were collated using thematic analysis and returned to participants in round two, allowing them to consider their colleagues' perspectives. Participants rated their agreement with the definition's accuracy using a Likert scale with the options “agree", “somewhat agree”, “unsure”, “somewhat disagree”, and "disagree." Responses were quantitatively analyzed using descriptive statistics to identify areas of consensus and divergence. Because consensus was achieved after two rounds, additional rounds were not warranted.

This study was approved by the City University of New York Institutional Review Board.

**Appendix Table 1. Participant characteristics and tests for heterogeneity by Rounds 1 and 2.**

| **Type** | **Category** | **Round 1 (n=34)** | **Round 2 (n=63)** | **Chi-square** | **p-value** |
| --- | --- | --- | --- | --- | --- |
| **Area of Expertise*** | AI & Machine Learning | 8 (8.25%) | 15 (8.24%) | 15.93 | 0.529 |
|  | Bioethics | 2 (2.06%) | 2 (1.1%) |  |  |
|  | Chronic disease management | 2 (2.06%) | 0 (0%) |  |  |
|  | Clinical Decision Making | 4 (4.12%) | 7 (3.85%) |  |  |
|  | Data Science & Analytics | 6 (6.19%) | 12 (6.59%) |  |  |
|  | Education | 6 (6.19%) | 7 (3.85%) |  |  |
|  | Electronic Health Records (EHR) | 5 (5.15%) | 3 (1.65%) |  |  |
|  | Global Health | 2 (2.06%) | 1 (0.55%) |  |  |
|  | Healthcare | 7 (7.22%) | 15 (8.24%) |  |  |
|  | Health Equity | 2 (2.06%) | 1 (0.55%) |  |  |
|  | Health Practice (all specialties) | 6 (6.19%) | 18 (9.89%) |  |  |
|  | Human Factors | 2 (2.06%) | 1 (0.55%) |  |  |
|  | Implementation Science | 1 (1.03%) | 2 (1.09%) |  |  |
|  | Informatics (all types) | 18 (18.56%) | 39 (21.43%) |  |  |
|  | Nursing | 6 (6.19%) | 9 (4.95%) |  |  |
|  | Quality & Saftey | 1 (1.03%) | 6 (3.3%) |  |  |
|  | Surgery (all types) | 0 (0%) | 2 (1.1%) |  |  |
|  | Other | 19 (19.59%) | 42 (23.08%) |  |  |
| **Hispanic or Latino origin** | Yes | 2 (5.88%) | 3 (4.76%) | 0.057 | 0.812 |
|  | No | 32 (94.12%) | 60 (95.24%) |  |  |
| **Race** | White or Caucasian | 22 (64.71%) | 37 (58.73%) | 1.98 | 0.852 |
|  | Black or African American | 1 (2.94%) | 5 (7.94%) |  |  |
|  | American Indian/Native American or Alaska Native | 1 (2.94%) | 1 (1.59%) |  |  |
|  | Asian | 8 (23.53%) | 13 (20.63%) |  |  |
|  | Other | 1 (2.94%) | 3 (4.76%) |  |  |
|  | Prefer not to say | 1 (2.94%) | 4 (6.35%) |  |  |
| **Education Level** | Bachelor’s degree | 1 (2.94%) | 2 (3.17%) | 0.004 | 0.950 |
|  | Graduate or professional degree (MA, MS, MBA, PhD, JD, MD, DDS etc.) | 33 (97.06%) | 61 (96.83%) |  |  |
| **Age** | 18-24 years old | 1 (2.94%) | 0 (0%) | 3.86 | 0.696 |
|  | 25-34 years old | 8 (23.53%) | 9 (14.52%) |  |  |
|  | 35-44 years old | 8 (23.53%) | 16 (25.81%) |  |  |
|  | 45-54 years old | 6 (17.65%) | 14 (22.58%) |  |  |
|  | 55-64 years old | 7 (20.59%) | 14 (22.58%) |  |  |
|  | 65+ years old | 4 (11.76%) | 9 (14.52%) |  |  |
|  | Prefer not to say | 0 (0%) | 1 (1.61%) |  |  |
| **Gender** | Male | 14 (41.18%) | 26 (41.27%) | 0.203 | 0.904 |
|  | Female | 19 (55.88%) | 36 (57.14%) |  |  |
|  | Prefer not to say | 1 (2.94%) | 1 (1.59%) |  |  |
| **Credentials** | CISSP | 0 (0%) | 1 (1.59%) | 14.76 | 0.738 |
|  | MD | 5 (14.71%) | 13 (20.63%) |  |  |
|  | PhD | 8 (23.53%) | 14 (22.22%) |  |  |
|  | DNP | 1 (2.94%) | 0 (0%) |  |  |
|  | PharmD | 2 (5.88%) | 2 (3.17%) |  |  |
|  | RN | 1 (2.94%) | 2 (3.17%) |  |  |
|  | MBI, MS | 1 (2.94%) | 0 (0%) |  |  |
|  | MD, MBI | 0 (0%) | 2 (3.17%) |  |  |
|  | MD, MS | 0 (0%) | 2 (3.17%) |  |  |
|  | MD, MSHI | 0 (0%) | 1 (1.59%) |  |  |
|  | MD, PhD | 1 (2.94%) | 2 (3.17%) |  |  |
|  | PhD, MS | 1 (2.94%) | 0 (0%) |  |  |
|  | PhD, RN | 8 (23.53%) | 14 (22.22%) |  |  |
|  | MD, MPH, MS | 0 (0%) | 1 (1.59%) |  |  |
|  | PhD, RN, MPH | 1 (2.94%) | 1 (1.59%) |  |  |
|  | RN, MSN, MBA | 1 (2.94%) | 0 (0%) |  |  |
|  | ANP, CRNA, CNS, RN | 1 (2.94%) | 0 (0%) |  |  |
|  | MD, PhD, MBA, MS, MPH | 0 (0%) | 1 (1.59%) |  |  |
|  | PhD, ANP, CRNA, CNS, RN | 1 (2.94%) | 2 (3.17%) |  |  |
|  | Other | 2 (5.88%) | 5 (7.94%) |  |  |
| **Area of Work** | Hospital/Health care system | 7 (20.59%) | 10 (15.87%) | 1.33 | 0.856 |
|  | Academics | 22 (64.71%) | 38 (60.32%) |  |  |
|  | Industry | 1 (2.94%) | 4 (6.35%) |  |  |
|  | Public sector | 2 (5.88%) | 5 (7.94%) |  |  |
|  | Other | 2 (5.88%) | 6 (9.52%) |  |  |

**Note: For area of expertise, participants identified up to three categories; thus, the sum of responses will be greater than the number of respondents.*
